# Clinical significance of resting and dobutamine stress myocardial work in cirrhotic cardiomyopathy

**DOI:** 10.1186/s44156-026-00136-0

**Published:** 2026-09-14

**Authors:** Jeyamani Ramachandran, Purendra Kumar Pati, Fadak Mohammadi, Majo Jospeh, Teresa Hecker, Mohamed Asif Chinnaratha, James Gunton, Kate Muller, Matthew Chapman, Richard Woodman, Alan Wigg

**Affiliations:** 1 Hepatology and Liver Transplant Medicine Unit, Flinders Medical Centre, Bedford Park, Adelaide, SA 5042 Australia; 2 College of Medicine and Public Health, Flinders University, Adelaide, Australia; 3 Department of Cardiology, Lyell McEwin Hospital, Adelaide, Australia; 4 School of Medicine, Adelaide University, Adelaide, Australia; 5 Department of Cardiology, Flinders Medical Centre, Adelaide, Australia; 6 Department of Gastroenterology, Lyell McEwin Hospital, Adelaide, Australia; 7 Department of Biostatistics, Flinders University, Adelaide, Australia

**Keywords:** Cardiac dysfunction, Liver failure, Stress echocardiography

## Abstract

**Background:**

Myocardial work (MW), an emerging measure of cardiac function, has not been assessed in cirrhotic cardiomyopathy (CCM) unlike global longitudinal strain (GLS).

**Aims:**

To evaluate MW in cirrhosis with transthoracic echocardiography (TTE) and dobutamine stress echocardiography (DSE); to study association of MW and CCM with a composite study outcome (one or more of acute kidney injury/hepatorenal syndrome, refractory ascites, cardiac failure, death); to assess the utility of cardiac biomarkers (NT-pro-BNP and cardiac troponin T) in the diagnosis of CCM; and to re-assess cardiac function at 12 months of LT.

**Methods:**

*Design*: Prospective study. *Participants*: Decompensated liver cirrhotic patients with ascites and those undergoing liver transplant (LT) assessment from January 2021 to August 2022 with a 12-month follow-up. *Study*: TTE- and DSE-reported systolic and diastolic parameters as well as GLS and MW indices were calculated.

**Results:**

96 patients, mean ± SD age 56 ± 11 years (58% male; 59% alcohol misuse) with Model for End-stage Liver Disease Sodium (MELD-Na) 17 ± 7 were studied. During the follow-up, 16 underwent LT; 53 developed the study outcome. CCM was diagnosed in 26 (27%), and it had no association with study outcome. MW was decreased in patients with CCM, and there was no augmentation of GLS or MW on DSE. MELD-Na. GLS, MW indices and septal E/e’ were higher in patients meeting the study outcome. MELD-Na and septal E/e’ were independent predictors of the study outcome. There was no association between biomarkers and CCM. Ten patients evaluated post LT demonstrated significant reductions in GLS and MW.

**Conclusions:**

MW was decreased in patients with CCM. Neither MW indices nor a diagnosis of CCM was predictive of the study outcome. Liver disease severity measured by MELD-Na score and E/e’ were independent predictors of the study outcome. Although MW is a load-independent measure, in this study, it did not add to the diagnosis CCM more than GLS. Further large-scale studies are required to establish the role of MW in evaluation of patients with liver cirrhosis.

## Introduction

Cirrhotic cardiomyopathy (CCM) is a type of chronic cardiac dysfunction observed in cirrhotic patients of any aetiology in the absence of a primary cardiac cause [1]. CCM is defined as systolic dysfunction (SysD) and/or diastolic dysfunction (DD) and is often associated with electrophysiological changes [2]. It remains subclinical in the vast majority but can become overt during stressful situations such as liver transplantation (LT), transjugular intrahepatic portosystemic shunt insertion and sepsis [3]. CCM has been shown to predispose to complications of cirrhosis such as hepatorenal syndrome, especially in conjunction with beta-blockers [4]. Even after successful LT, it has been shown to increase risk of cardiac failure, graft dysfunction and mortality [5].

The original diagnostic criteria for CCM defined SysD using left ventricular ejection fraction (LVEF) < 55% alone [2]. Global longitudinal strain (GLS) measurement by speckle tracking echocardiography (STE) is included in the updated CCM diagnostic criteria to detect subclinical myocardial dysfunction [2]. The overall prevalence of CCM based on these two criteria showed no significant difference – 67% with the old criteria vs. 55% with the new criteria in a recent study that compared old and new CCM criteria in a cohort of patients with cirrhosis [6]. However, the systolic dysfunction was significantly more prevalent at 55% using the new criteria compared to 13% with the old criteria [6]. 

GLS is less dependent on afterload than LVEF. Despite this, GLS measurements have yielded conflicting results in cirrhotic patients [7–10]. Whilst the new criteria of CCM does not include dobutamine stress echocardiography (DSE) in its formal diagnostic criteria, impaired contractile reserve is highlighted as a potential marker of CCM [2]. 

Myocardial work (MW) is a novel echocardiographic measure of myocardial performance independent of afterload, which is decreased in cirrhosis and hence may be a more accurate assessment of true contractile function in these patients [11, 12]. MW is increasingly used to diagnose and prognosticate cardiac dysfunction comprehensively in many types of cardiomyopathy [12]. MW is defined as the amount of energy required to eject and propel a given volume of blood through vasculature and directly represents myocardial oxygen consumption. However, pressure–volume loop is an invasive and time-consuming procedure, whilst MW is a non-invasive method of estimating myocardial funtion, and it incorporates afterload, myocardial oxygen consumption and glucose metabolism [13]. Therefore, it provides a more precise assessment of myocardial function than deformation parameters such as LVEF and GLS. It is calculated by a special echocardiographic software combining GLS with simultaneous blood pressure (BP) measurements.

In this prospective study of patients with advanced cirrhosis, we hypothesize that MW will be a more accurate and less afterload dependent parameter in diagnosing CCM. The primary aim of the study was (i) to evaluate global cardiac function in decompensated cirrhosis (DC) using advanced echocardiographic parameters at rest and with DSE, (ii) report on prevalence of CCM and MW indices in cirrhosis and (iii) to study their association with development of a composite clinical outcome including acute kidney injury/hepatorenal syndrome (AKI/HRS), refractory ascites, cardiac failure and death over the subsequent 12 months. The secondary aims were (iv) to assess the diagnostic utility of cardiac biomarkers (NT-pro-BNP and cardiac troponin T) in CCM, and (v) to reassess cardiac function at 12 months of successful LT.

## Methods

This is a prospective cohort study conducted in the hepatology units of two major tertiary hospitals in South Australia from January 2021 to August 2022.

Inclusion criteria:

Consenting patients ≥ 18 years, with a diagnosis of cirrhosis (based on clinical, laboratory and ultrasonographic features) with ascites and/or past history of ascites on diuretics, those with hydrothorax, and those undergoing LT assessment with or without ascites.

Exclusion criteria: Cirrhotic patients with the following conditions were excluded.

Ongoing sepsis on inotropes; Ongoing grade 2–4 hepatic encephalopathy; Known coronary artery disease; Other known cardiac diagnoses such as valvular heart disease, dilated, hypertrophic and restrictive cardiomyopathy; Uncontrolled systemic hypertension;. Contraindications to dobutamine, including: Previous sensitivity to dobutamine and previous documented Ventricular Tachycardia or Ventricular Fibrillation.

Unit 1: This LT unit recruited patients throughout the study period allowing for COVID-related restrictions on elective services that affected recruitment. Of the 146 patients fulfilling inclusion criteria, 61 patients (29 with pre-existing heart disease; 17, unwilling to participate; 15 due to unstable liver disease or COVID-related causes) were excluded. Among the 85 patients who consented, 80 were included, after excluding 5 poor-quality echocardiographic images.

Unit 2: A non-LT unit, recruited patients from March to December 2021. Among the 43 patients admitted with cirrhotic ascites, 25 consented to the study; 18 patients (8 with pre-existing heart disease; 5, refusals to undergo echocardiogram; 5 due to infections and/or hepatic encephalopathy) were excluded. Among the 25 consented, only 16 patients underwent transthoracic echocardiography (TTE) and only 10 had DSE.

**Fig. 1:**
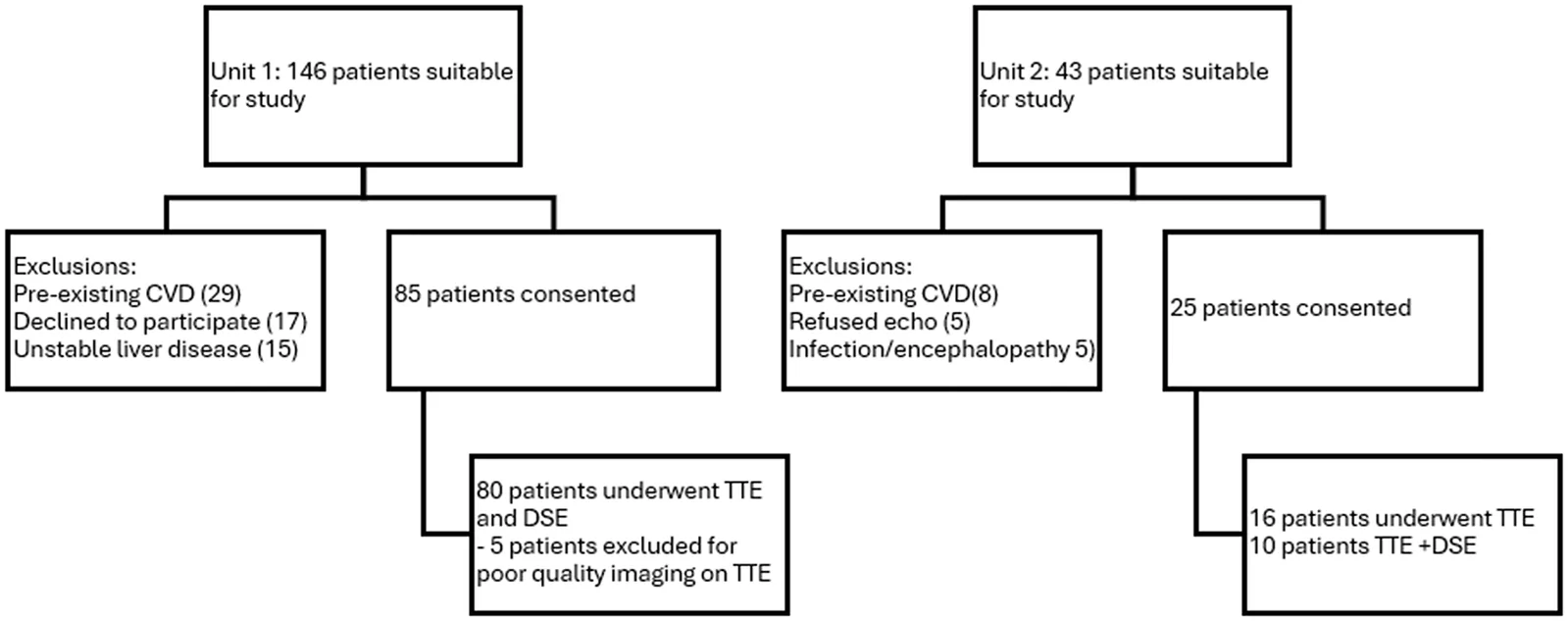
Flow chart of included and excluded patients. CVD, cardiovascular disease; DSE, dobutamine stress echocardiography; TTE, trans-thoracic echocardiography

Patient recruitment details are summarised in Fig. 1.

Sample size justification: With a sample size of 78 subjects, the study was to have 80% power to detect a 30% reduction in the composite outcome for a 1 SD increase in the GWI using Cox regression, assuming an *R*-squared of 30% with other variables and a two-sided type 1 error rate of alpha = 0.05.

Ethics approval: The study was approved by the Institutional Ethics Committee HREC/20/SAC/211. The study was conducted in full conformance with principles of the “Declaration of Helsinki”, Good Clinical Practice (GCP), the National Statement on Ethical Conduct in Human Research (NHMRC, 2007), Australian Code for the Responsible Conduct of Research (2007) and within the laws and regulations Australia.

Procedure: After a written consent, the following data were collected: clinical profile (liver disease aetiology, cirrhotic complications and severity using Model for End-stage Liver Disease Sodium (MELD-Na) score, use of beta-blockers) and cardiac biomarker levels.

### Echocardiography

TTE and DSE were performed. Standard echocardiographic data including left ventricular ejection fraction (LVEF), ventricular dimensions and wall thickness, mitral inflow E, A, E/A ratio, mitral inflow deceleration time and, if tricuspid regurgitation (TR) was present, then TR velocity and gradient study were recorded. Mitral annulus velocity (e’) was calculated; E/e’ ratio (E, transmitral early peak velocity) and left atrial volume index (LAVI) were also calculated. Using speckle tracking technique, from apical 4 C, 2 C and long axis, GLS was calculated from pre-specified frame rates (70 fps, frames per second), and simultaneously BP was recorded.

### Myocardial work

Using special automated software, MW including GWI, GCW, GWE and GWW were calculated from strain echocardiography combined with concurrent brachial BP at rest and during DSE as follows Myocardial work calculations were performed using the GE Echopac 203 analysis platform. Once the standard strain curves (GLS) were obtained, myocardial work analysis was initiated. The software displays the timings of mitral valve and aortic valve opening and closure in relation to the ECG trace. These timings can be manipulated manually if the software’s automatic event markers do not accurately correspond with the visual assessments of valve motion. Systolic and diastolic cuff blood pressures, obtained at the time of image acquisition, were entered into the analysis program.Once inputs are confirmed and accepted the myocardial work analysis package automatically generates the GWI, GCW, GWE and GWW in the form of overall values, segmental bullseye plots and pressure - strain loops [14]. 

Four indices are calculated as a part of MW measurement including:

1. Global work index (GWI) mmHg%: GWI represents global myocardial performance, especially efficiency of cardiac pumping action during systole. Normal range: 95% CI Females: 1310–2538, Males: 1270–2428,
2. Global constructive work (GCW) mmHg%: GCW represents amount of energy spent effectively to pump blood out of heart (i.e. cardiac output) and is a summation of positive work during systole and negative work during isovolumetric relaxation. Normal range: 95% CI Females: 1543–2924, Males: 1650–2807,
3. Global wasted work (GWW) mmHg%: GWW reflects both negative work (segment lengthening) during systole and positive work (segment shortening) during isovolumetric relaxation. confidence limits of normality ± SE.: Females: 239 ± 39, Males: 238 ± 33,
4. Global work efficiency (GWE) %: GWE represents the ratio of constructive work divided by sum of constructive and wasted work. Confidence limits of normality ± SE: Females: 91 ± 1, Males: 90 ± 1.6.

DSE was done to assess contractile reserve and to rule out ischemia, and it was done on the same day or within the next 2 weeks using a 12-lead ECG for continuous monitoring. Dobutamine (250 mg/100 ml concentration) was infused for 3 min each at 10, 30 and 50 µg/kg/min. The target heart rate was 85% of predicted maximum heart rate (220 minus age). Echocardiographic images from the parasternal and apical windows were obtained at rest and after each 3-min dose, with final images at peak heart rate. For GLS and MW an extra image of apical long axis was required. BP was monitored at 3-min intervals throughout and into recovery; MW was calculated.

### Definition of CCM

CCM was defined according to the consortium criteria (2019) as the presence of SysD and/or DD as follows: [2]

I. SysD was defined as LVEF < 50% and/or absolute GLS < 18%.
II. DD was diagnosed when three of the four criteria were met:
  1. Septal (e′) < 7 cm/s or lateral e′ velocity < 10 cm by tissue Doppler.
  2. E to e′ ratio > 14 (using average e′) or 15 (using medial e′).
  3. LAVI > 34 ml/m^2^.
  4. TR velocity > 2.8 m/s.

### Outcome definition

The primary objectives were to report on the MW in the study cohort, measure prevalence of CCM as per the current criteria and study the association of CCM and MW with the study. outcome. The study outcome was a composite clinical outcome including acute kidney injury/hepatorenal syndrome (AKI/HRS), refractory ascites, cardiac failure and death over the subsequent 12 months.

Follow-up for development of clinical endpoints was performed using electronic medical records. Patients were censored at LT, death or up to 12 months from recruitment, whichever happened earlier. Patients undergoing LT were approached for repeat TTE 12 months after LT. The secondary outcomes were to evaluate cardiac biomarkers (NT-pro-BNP and cardiac troponin T) in CCM, and to re-assess cardiac function at 12 months of successful LT whenever possible.

### Statistical methods

Statistical analysis was conducted using SPSS IBM Statistics version 29 (IMB Corp., Armon NY, USA). Continuous data were presented as mean (standard deviation, SD) or median (interquartile range, IQR) depending on the normality of distribution. Categorical variables are presented as numbers and percentages. Difference in estimated marginal mean difference in myocaridal indices between baseline and repeated values during follow-up were obtained using repeated measures of ANOVA. Rank correlations were assessed using Spearman’s rho. Univariate analysis of clinical, echocardiographic and demographic features on the study outcome was assessed using either independent *t*-test or chi-squared test as appropriate. Significant predictors from the univariate analysis (*p* < 0.05) were included in multivariate Cox regression analysis. A two-sided type 1 error rate of alpha = 0.05 was used for assessing statistical significance.

## Results

Ninety-six cirrhotic patients were studied over a median (IQR) follow-up period of 365 (209) days. Their mean ± SD age was 56 ± 11 years; 58% were male; 59% of cirrhosis was alcohol misuse related; 16 patients were on beta-blockers; 47 (49%) patients had Child’s class C cirrhosis, 41 (43%) Child’s class B and 8 (8%) Child’s class A. Baseline clinical and biochemical parameters are listed in Table 1.

**Table 1:** Demographic, clinical and laboratory features according to composite study outcome (*N* = 96)

Parameters	Entire study cohort (*N* = 96)	Patients experiencing study outcome (*N* = 53)	Patients not experiencing study outcome (*N* = 43)	*p*-value*
Age (years)	56 (11)	57 (11)	56 (11)	0.544
MELD-Na score	17 (7)	20 (7)	14 (6)	< 0.001
Alcohol as aetiology	57 (59%)	33 (58%)	24 (42%)	0.522
Systolic BP, mmHg	118 (19)	120 (20)	117 (19)	0.549
Diastolic BP, mmHg	63 (13)	61 (13)	65 (12)	0.076
Heart rate, bpm	80 (15)	83 (15)	77 (15)	0.044
Prolonged QT interval (> 440 ms, males; 460 ms, females), *n* = 91	51 (56%)	31 (60.8%)	20 (39.2%)	0.066
Creatinine, µmol/l (median, IQR)	73 (41)	86 (39)	69 (22)	0.008
Sodium, mEq/l	135 (5)	134 (4)	137 (5)	< 0.001
Bilirubin, µmol (median, IQR)	50 (71)	96 (108)	69 (83)	0.174
Troponin T, ng/ml (median, IQR)	14 (18)	51 (108)	15 (18)	0.034
BNP, pg/ml (median, IQR)	356 (322)	399 (517)	303 (661)	0.438

Data are mean (SD) unless otherwise indicated

*Calculated using independent *t*-test Mann–Whitey test, or chi-squared test as appropriate

### MW and markers of SysD

Echocardiographic parameters of the study cohort including MW at baseline and DSE are listed in Table 2.

**Table 2:** Univariate analysis of echocardiographic features by study outcome

Parameters	Entire study cohort (*N* = 96)	Patients experiencing outcome (*N* = 53)	Patients not experiencing outcome (*N* = 43)	*p*-value*
Rest LVEF, %	68.8 (6)	70.1 (6)	67.2 (7)	0.030
Rest GLS, %	–20.4 (2)	–20.5 (2)	–19.2 (2)	0.008
Rest GWI (mmHg%)	1892.3 (402)	1914.3 (409)	1731.1 (408)	0.036
Rest GCW (mmHg%)	2236.7 (440)	2238.0 (467)	2103.8 (405)	0.145
Rest GWW (mmHg%)	92.7 (55)	97.1 (49)	108.4 (59)	0.389
Rest GWE (mmHg%)	95.3 (3)	94.9 (2)	94.3 (3)	0.214
Septal e’(cm/s) (median, IQR)	8 (2)	7 (1)	8 (2)	0.494
Lateral e’ (median, IQR)	11 (5)	10 (3)	3 (1.7)	0.308
Septal E/e’ ratio (median, IQR)	10.8 (4)	12.1 (3)	9.5 (3)	< 0.001
Average E/e’ ratio (median, IQR)	8.8 (4)	10.4 (3)	8.0 (3)	< 0.001
LAVI	35.5 (18)	40.5 (15)	34.0 (11)	0.016
TR gradient	2.6 (0.4)	2.7 (0.4)	2.42 (0.3)	0.022
DSE parameters	*N* = 72	*N* = 39	*N* = 33	
Peak LVEF	78.6 (6)	79.4 (5)	77.7 (6)	0.214
Peak GLS, % (*n* = 72)	–19.5 (3)	–20.4 (2)	–18.5 (3)	0.004
Peak GWI (mmHg%)	1480.0 (431)	1574.7 (394)	1368.1 (452)	0.042
Peak GCW (mmHg%)	1891.7 (117)	1958.7 (427)	1812.6 (496)	0.184
Peak GWW (mmHg%)	192.3 (117)	164.6 (97)	225.0 (131)	0.033
Peak GWE (mmHg%)	89.2 (6)	90.7 (5)	87.3 (6)	0.015

Data are mean (SD) unless otherwise indicated

*From independent *t*-test or Mann–Whitey test as appropriate

As defined by a low GLS < 18%, 16 patients (17%) had SysD, none with LVEF < 50%. Mean MELD-Na was significantly lower in patients with SysD than those without (13.8 ± 7.5 vs. 18.1 ± 6.6, *p* = 0.022). In univariate analysis, GLS at rest and peak GLS (GWI at rest and peak GWI), peak GWW and GWE were all associated with the composite study outcome and included in in multivariate Cox regression. All parameters except rest GWW were lower in those with CCM compared to those without, but there was no difference noted on DSE (Table 3).

**Table 3:** Myocardial work and Cirrhotic cardiomyopathy (*N* = 91)

Parameters	CCM (*N* = 26)	No CCM (*N* = 65)	*p*-value*
GLS (%)	–18.1 (2)	20.1 (2)	< 0.001
GWI (rest) (mmHg%)	1425.5 (303.6)	1915.1 (393.5)	< 0.001
GCW (rest) (mmHg%)	1851.6 (336.8)	2248.7 (435.1)	0.020
GWW (rest) (mmHg%)	134.6 (66.6)	95.2 (55.2)	0.014
GWE (rest) (%)	92.4 (2.6)	95.1 (2.3)	< 0.001
DSE parameters	CCM (16)	No CCM (56)	
GLS peak (mmHg%)	–18.6 (3)	–19.8 (3)	0.133
GWI peak (mmHg%)	1400.38 (483)	1502.73 (417)	0.407
GCW peak (mmHg%)	1808.94 (552)	1915 (436)	0.421
GWW peak (mmHg%)	218.88 (127)	184.64 (114)	0.306
GWE peak (%)	87.25 (7)	89.71 (5)	0.134

Data are mean (SD) unless otherwise indicated

^*^From independent *t*-test or Mann–Whitey test as appropriate

CCM: Cirrhotic cardiomyopathy

### DD

Applying the CCM consortium diagnostic criteria, 9 (9.5%) of 96 patients had DD. The presence of DD was not associated with the study composite outcome (*n* = 9, 7 (775%) vs 2 (22%); *p*0.2). However, as described below, when individual parameters were analysed, septal and average E/e’, LAVI and TR Vmax (maximal TR velocity) were associated with the study outcome on univariate analysis. In multivariate analysis, septal E/e’,was associated with the study outcome.

### MW in CCM

Twenty-six (27.4%) patients had CCM according to the consortium criteria; 16 of them had SysD and 12 had DD. Two patients had both SysD and DD. Comparison of MW between patients with and without CCM are shown in Table 3. Interestingly, all MW indices including GWI, GCW and GWE were significantly decreased in CCM except for increased GWW. There was no clinical evidence of cardiac failure in patients who had echocardiographic evidence of CCM. There was no difference in the occurrence of study outcomes in those with and without CCM (*n* = 15/26, 57.7% vs. *n* = 11/26, 42.3%; *p* = 0.523).

### MW on DSE

GLS and MW measured at various stages of DSE are listed in Table 4. Among the 72 patients with paired GLS analysis before and after DSE, 54 (75%) did not show any augmentation on DSE suggesting impaired systolic function. Table 4 depicts the MW indices, LVEF and GLS at baseline and 10, 30 µg/kg/min and peak doses of dobutamine. Overall, compared to baseline values, there were increases in LVEF at 10, 30 µg/kg/min and peak, decreases in GLS at 10 µg/kg/min and peak, decreases in GCW and GWI at 30 µg/kg/min and peak, decreases in GWE at dobutamine 30 µg/kg/min and peak, and increase in GWW at 10, 30 µg/kg/min and peak.

**Table 4:** Comparison of global cardiac function at rest and after dobutamine stress echo (*N* = 72)

Parameters	Baseline	Dobutamine		
		10 µg/kg/min	30 µg/kg/min	Peak
LVEF %(*n* = 74)	69.8 (5.9)	72.6 (5.8) Δ = 2.80 (1.67 to 3.92) *p* = < 0.001	76.9 (5.5) Δ=–7.14 (5.81 to 8.47) *p* = < 0.001	78.5 (5.6) Δ = 8.71 (7.23 to 10.18) *p* = < 0.001
GLS (%) (*n* = 72)	–20.5 (2.3)	21.4 (2.7) Δ=-0.85 (–1.29 to − 0.41) *p* = 0.002	21.0 (2.8) Δ = 0.48 (–0.96 to 0.00) *p* = 0.050	19.7 (2.7) Δ = 0.79 (0.24 to 1.35) *p* = 0.005
GWI (mmHg%)	1893.5 (398)	1896 (439) Δ = 2.06 (–56.3 to 60.5) *p* = 0.945	1739 (437) Δ=–154.2 (–240.7 to − 67.8) *p* < 0.001	1482 (435) Δ=–411.7 (–499.9 to − 323.6) *p* < 0.001
GCW (mmHg%)	2235 (423)	2234 (479) Δ = 0.27 (–69.7 to 69.2) *p* = 0.994	2091 (486) Δ = 144.3 (246.7 to − 41.9) *p* = 0.005	1897 (471) Δ = 337.5 (–443.6 to − 231.3) *p* = < 0.001
GWW (mmHg%)	92.9 (54.6)	136.3 (184.6) Δ = 43.4 (–1.9 to 88.7) *p* = 0.060	154.8 (92.5) Δ=–61.8 (40.6 to 83.0) *p* = < 0.001	187.3 (117.5) Δ=–94.4 (64.9 to 123.8) *p* = < 0.001
GWE (%)	95.2 (2.1)	94.2 (2.6) Δ=–1.01 (–1.67 to − 0.35) *p* = 0.003	91.8 (4.4) Δ=–3.39 (–4.39 to − 2.38) *p* = < 0.001	89.5 (5.6) Δ=–5.72 (–7.03 to − 4.41) *p* = < 0.001

Δ = Difference (95% CI) in estimated marginal mean from baseline obtained using repeated measures ANOVA

*p*-values represent comparisons between baseline and peak DSE parameters using repeated measures ANOVA

### Study outcome

During the median (IQR) follow-up period of 365 (158–265) days, a total of 53 (55.8%) patients experienced the study composite outcome; 28 had HRS/AKI; 41 had refractory ascites and 16 patients died. No patient developed clinical evidence of heart failure. In univariate analysis using *t*-tests, MELD-Na, troponin, LVEF at rest, GLS at rest and peak, GWI at rest and peak, peak GWW, peak GWE, septal and average E/e’, LAVI and TR Vmax (maximal TR velocity) were associated with the study outcome. In multivariate Cox regression of the composite outcome, higher MELD-Na and higher E/e’ were independent predictors (Table 5). All included variables met the supremum test for the proportional hazards (*p* > 0.05). Subgroup analysis of patients with refractory ascites outcome revealed that severity of liver disease measured by MELD Na (mean difference 4.12, p = 0.003), higher septal E/e’ (mean difference 2.38, p < 0.001) average E/e’ (mean difference 2.17, p < 0.001) and peak GLS (mean difference 2.25%, p < 0.001) were signficantly different on univariate analysis. No significant differences were observed in age, blood pressure, troponin T, BNP, resting LVEF, peak LVEF, or most myocardial work indices. In multivariate analysis, MELD Na was the only independent predictor of refractory ascites. In the 28 patients with HRS/AKI had higher MELD Na ((mean difference 3.98, *p* = 0.010), significantly higher septal E/e’ (mean difference 2.11, *p* = 0.004) and average E/e’ (mean difference 2.05, *p* = 0.002) were signficant on univariate analysis. In multivariate analysis, MELD Na was the only independent predictor of HRS/AKI.

**Table 5:** Hazard ratios (95% CI) for the composite outcome from multivariate analysis

Parameters	HR (95% CI)	*p*-value
MELD-Na	1.076 (1.018–1.136)	0.009
Troponin	1.002 (0.999–1.004)	0.215
LVEF rest	1.013 (0.942–1.090)	0.732
GLS rest	0.911 (0.666–1.245)	0.558
GLS peak	1.068 (0.854–1.336)	0.566
GWI rest	1.000 (0.998–1.002)	0.977
GWI peak	1.000 (0.999–1.002)	0.853
GWW peak	0.995 (0.985–1.005)	0.305
GWE peak	0.967 (0.768–1.217)	0.773
Septal E/e’	1.179 (1.037–1.340)	0.012
LAVI	1.001 (0.970–1.033)	0.947

*p*-value obtained from Cox proportional hazards regression

### Correlation between echocardiographic parameters, cardiac biomarkers and MELD-Na

There was a moderately strong correlation between MELD-Na and resting GLS, BNP, troponin and a weak correlation with rest GWI and LAVI as listed in Table 6.

**Table 6:** Correlation between MELD-Na, echocardiographic parameters and cardiac biomarkers

MELD correlation with	Spearman’s coefficient	*p*-value
BNP	0.342	< 0.001
Troponin	0.338	< 0.001
LVEF	0.193	0.060
Peak LVEF	0.063	0.195
Resting GLS	0.343	< 0.001
Peak GLS	0.205	0.084
Rest GWI	0.230	0.027
Peak GWI	0.202	0.088
Peak GWW	–0.151	0.204
Peak GWE	0.232	0.060
Septal E/e’	0.169	0.101
LAVI	0.255	0.013

### LT

Forty-four patients were assessed for LT, and 16 underwent LT during the study follow-up; 7 of 16 patients (44.0%) had echo evidence of CCM (3, SysD; 4, DD), and none of them had any cardiac events in the perioperative or post-LT follow-up period. Ten patients had paired echoes and analyses of MW after 12 months LT, as shown in Table 7. Significant improvements in hyperdynamic circulation, defined by reductions in LVEF, GLS, MW and improvements in diastolic function defined by LAVI were demonstrated.

**Table 7:** Comparison of pre-LT and post-LT echoes (*N* = 10)

Parameters	Pre-LT	Post-LT	*p*-value
Systolic BP	127 (21)	116 (16)	0.25
Diastolic BP	61 (15)	70 (12)	0.25
Heart rate	77 (14)	70 (10)	0.16
LVEF	67.4 (6)	60.5 (9)	0.036
GLS	21.1 (2)	16.8 (3)	0.009
GWI	2101 (334)	1437 (324)	< 0.001
GCW	2466 (481)	1771 (354)	< 0.001
GWW	100 (51)	95 (37)	0.385
GWE	95 (2)	93 (2)	0.003
LAVI	49 (11)	29 (6)	< 0.001
Septal e’(cm/s)	0.08(0.02)	0.06(0.02)	0.12
Septal E/e’ ratio	12.2(3.2)	10.7(3.2)	0.15

Data are mean (SD)

### Serum biomarkers and CCM

Median (IQR) troponin was 13.5 (7.0–24.0) ng/ml and BNP was 189 (53.0–374.0) pg/ml. There was no significant association between these values and SysD, DD or CCM. However, troponin was significantly elevated in those who met the composite study outcome (median 19.0 vs. 10.0; *p* < 0.001). However, when adjusted for other parameters in multivariate Cox regression analysis, it was not a predictor of the study outcome.

## Discussion

MW is increasingly used to diagnose and prognosticate cardiac dysfunction in many types of cardiomyopathy (including ischemic, non-ischemic dilated, hypertrophic and restrictive cardiomyopathy, as well as cardio oncology) in a comprehensive manner, non-invasively [12]. This study addressed the lack of data on MW in CCM and investigated global cardiac function in cirrhosis defining CCM with advanced echocardiographic methods at rest and with dobutamine stress echocardiography (DSE) and reported its clinical significance in relation to the development of a composite study outcome.

The prevalence of CCM in our study measured using the 2019 consortium criteria was 27%. This is very similar to the recent finding of 17% as per new criteria [15]. In patients with CCM, MW indices were all decreased except GWW (representing wasted work) that was increased.However, there was no association of decreased MW or CCM with development of composite study outcome. This is similar to the findings of prior studies that showed no association between major cardiac events and a diagnosis of CCM as per 2019 criteria [15]. On the contrary, patients experiencing the study outcome had higher GLS and GWI in our study. DD measured by E/e’ and liver disease severity assessed by MELD-Na were independent predictors of the study outcome.

In ischemic heart disease, athletes heart and cardiac amyloidosis, MW was found to be more sensitive than GLS in diagnosis and prediction of all-cause mortality [12]. However, MW parameters did not contribute significantly beyond GLS in diagnosing CCM. This observation mirrors findings in chemotherapy-related cardiac dysfunction, where MW also failed to demonstrate superiority over GLS [12]. 

The higher GLS and GWI noted at rest in patients experiencing the composite study outcome compared to those without, likely represented a state of hyperactive myocardium and a higher cardiac output state in advanced cirrhosis. This also explained the lower GWW and increased GWE noted in these patients representing more efficient work and less wasted work. Notably, neither GLS nor any of the MW indices were independent predictors of the study outcome.

While the new definition of CCM defined a cut-off of 18% GLS for SysD, we observed a lower GLS in patients with less severe liver disease (based on lower MELD-Na scores) and vice versa. In cirrhotic patients GLS measurements have yielded conflicting results. Earlier studies reported lower GLS values in cirrhotic patients (–18% vs. − 20%; *p* < 0.001) compared to healthy controls [7, 8]. This has been confirmed in a large meta-analysis examining 802 cirrhotic patients and healthy controls demonstrating a significantly lower GLS in the cirrhotic cohort (–19.8 vs. − 21.6, *p* < 0.001) [10]. However, these findings were not reported in association with any clinical outcomes. In contrast, more recent studies have demonstrated higher GLS in cirrhosis consistent with a hypercontractile state (–24.2 ± 2.7% in cirrhosis vs. − 18.6 ± 2.2% in healthy controls; *p* < 0.001) [9, 10]. 

A recent retrospective study that compared GLS in compensated cirrhosis (CC) and DC demonstrated results similar to ours with a higher GLS in DC [16]. However, both low and high GLS were associated with reduced survival when these patients were followed up over 3 years. Impaired GLS (< 20.5%) was shown to predispose to post-LT congestive heart failure (CHF) and coronary artery disease [17]. Interestingly, Pagourelias et al. measured speckle tracking parameters in 77 cirrhotic patients and found that GLS did not differ across the three Child–Pugh classes (–20.1%, − 21.3% and − 21.0% for Child–Pugh A, B and C, respectively) [18]. A similar lack of differences between GLS across the three Child–Pugh classes were reported by Hammami et al. in their prospective evaluation of 80 cirrhotic patients [19]. The heterogeneity between GLS and liver disease severity across various studies could be due to the differences in the state of myocardium at the time of measurement. Serial measurement of GLS with a reducing trend is more likely to be reflective of SysD than a single value. A small longitudinal study showed no progression of cardiac abnormalities in patients with stable cirrhosis over 2 years compared to those with acute decompensation (AD), which was associated with low cardiac index [20]. However, GLS did not change in patients who developed decompensation or death.

Impaired contractile reserve is cited as a potential marker of CCM although not included in the 2019 cosortium criteria [2, 21]. Our study evaluated the performance of GLS and MW with DSE and examined their association with important clinical outcomes in cirrhosis, which has not been previously reported. There was no augmentation of GLS and MW, and this lack of inotropic response was noted without any clinical association. However, a study of MW assessed during DSE in normal patients revealed no incrementation similar to our findings [22]. Due to lack of significant differences in MW and GLS in patients with CCM on DSE, we agree with the updated CCM criteria that has excluded DSE. A small study that compared cirrhosis and controls showed a blunted GLS response to DSE in cirrhosis [10]. Sampaio et al. showed an impaired GLS increase in patients as compared to controls during low (median increase of 6.6% vs. 28.6%; *p* < 0.001) and intermediate dose dobutamine (median increase of 2.6% vs. 12.6%; *p* = 0.016) on magnetic resonance imaging [23]. The need to intergrate myocardial contractile reserve in evaluation for CCM is demonstrated in a recent study. Impaired cardiac reserve defined as inability to increase cardiac output by > 25% on dobutamine infusion was noted in 33% of patients undergoing LT and was associated with MACE [24]. 

DD was more often seen in patients meeting the study outcome, although it did not reach statistical significance. Prior studies have demonstrated an association with DD and increased risk of rejection, death and graft failure in LT recipients [25]. The 4 patients with DD in our study who underwent LT did not experience any adverse events after LT. Karagiannakis et al. reported an independent predictive role of DD in advanced cirrhosis, whereas Sonny et al. did not find any clinical impact of the DD diagnosed before LT [26, 27]. A strong association between DD and HRS development and mortality was demonstrated by Ruíz-del-Árbol et al.; similar to our study, MELD-Na and E/e’ were independent predictors of mortality [4]. 

There was an independent association between MELD-Na and clinical outcomes rather than any of systolic echocardiographic parameters including MW. Similarly, there was no correlation between cardiac biomarkers and the composite clinical outcome in this study. This confirmed the findings previously reported from our unit showing lack of impact of cardiac dysfunction on survival of patients assessed for LT [28]. This is similar to findings by Sampaio et al. who did not find any association with SysD or DD and medium-term mortality in cirrhosis [29]. 

Our study demonstrated significant improvements in hyperdynamic circulation defined by reductions in LVEF, GLS, MW and improvements in diastolic function defined by LAVI at 12 months after successful LT. Similar findings were reported by Kim et al. with high GLS before LT that normalised 1-year post LT (from − 24.9 ± 2.4% to − 20.6 ± 3.4%; *p* < 0.001) [9]. In contrast, improved GLS (from − 18.5 ± 2.6 to − 20.8 ± 2.0) was reported by Chen et al. [7] The normalisation or reduction of GLS and MW post LT in our study confirmed reversal of the hypercontractile state of myocardium in response to systemic vasodilatation in cirrhosis and the pathophysiology of CCM [30, 31]. 

### Limitations and strengths

Afterload independence makes MW preferable to load-dependent parameters such as LVEF and GLS to measure cardiac function. However, its measurement is dependent on good-quality echoes, sinus rhythm and absence of aortic stenosis or peripheral vascular disease. Although primary cardiac causes were excluded in this study by history and echocardiography, CT coronary angiography might have detected asymptomatic coronary disease as a cause of DD. DSE evaluated only SysD, whereas diastolic markers were not studied on DSE. Cardiac output was not measured, as the premise of the study was to evaluate global cardiac function in a non-invasive manner. A larger study sample size and more post-LT follow-up echocardiograms would have provided robustness to the interesting pre- and post-LT comparison findings. CCM was diagnosed only by echocardiographic criteria. Correlation with cardiopulmonary exercise testing (CPET) and measurement of peak oxygen consumption might have identified patients with subclinical cardiac dysfunction.

Despite these limitations, the many strengths of this first study of MW in CCM include a well-calculated sample size, prospective design, evaluation with DSE and post-LT evaluation.

## Conclusions

This prospective study of MW indices using advanced echocardiography in a cohort of advanced cirrhosis revealed decreased MW in patients with CCM. Neither this nor echocardiographic evidence of CCM had any impact on clinical course before or after LT. However, the increased GLS and MW observed in patients with study outcome reflective of hyperdynamic circulation need to be explored in larger prospective longitudinal studies. The study also cast doubts on the veracity of the GLS cut-off proposed in the updated CCM criteria. DD was associated with the study composite outcome in addition to the severity of cirrhosis. LT was successful in reversing the hypercontractile myocardium, and thus LT seems to be the only effective therapeutic modality for CCM. Further large, multicentre, longitudinal studies are warranted to improve our understanding of the role of MW, its correlation with CPET findings and its clinical significance in cirrhosis.

## Data Availability

Data, analytical methods and study materials will be made available if necessary after clearance from local ethics committee.
